# Divergent patterns of confrontation with death using the Anticipated Farewell to Existence Questionnaire (AFEQT): a cross-sectional comparative study of four samples with increasing proximity to death

**DOI:** 10.1186/s12904-021-00818-y

**Published:** 2021-08-08

**Authors:** Juan Valdés-Stauber, Ursula Stabenow, Jakob Böttinger, Sarah Kramer, Reinhold Kilian

**Affiliations:** 1 Department of Psychiatry and Psychotherapy I, University of Ulm, Ulm, Germany; 2 Zentrum für Psychiatrie Südwürttemberg, Weingartshofer Strasse 2, 88214 Ravensburg, Germany; 3 Department of Psychosomatic Medicine and Psychotherapy, Diakonissenkrankenhaus Karlsruhe Rüppurr, Diakonissenstrasse 28, 76199 Karlsruhe, Germany; 4 Department of Psychiatry and Psychotherapy II, University of Ulm, Ulm, Germany; 5 Bezirkskrankenhaus Günzburg, Lindenallee 2, 89312 Günzburg, Germany

**Keywords:** Death, Dying, Terminal care, Philosophical anthropology, Anticipatory farewell to existence

## Abstract

**Background:**

Based on the concept of “*Daseinsverabschiedung*”, an anthropological theory of “Anticipated Farewell to Existence” (AFE) was suggested on the basis of six grounding dimensions: selfhood, interpersonality, temporality, corporeality, worldliness, and transcendence, which are activated in a genuine manner facing death. The purpose of the study is to quantitatively compare the extent of confrontation with death between dying people in palliative care and those in other stages of life by means of the Anticipated Farewell to Existence Questionnaire” (AFEQT), based on these dimensions.

**Methods:**

The sample (N = 485) consists of dying individuals in palliative wards and hospices (n = 121); old people living in nursing homes not suffering from a mortal disease (n = 62); young adults (n = 152), and middle-aged adults (n = 150). The design is cross-sectional and analytical. The relevance of anticipated farewell to existence was measured by means of the AFEQT. The internal consistency of the AFEQT was assessed using Cronbach’s alpha and convergent validity by means of dimensions of the Life Attitude Profile-Revised (LAP-R). Differences between groups and associations with control variables were estimated by means of multiple regression models, including propensity scores.

**Results:**

Cronbach’s alpha for AFEQT was > 0.80 for the whole test and all subsamples, but < 0.70 for most dimensions in dying people. Correlations between each dimension and corresponding two factors was almost overall r > 0.80, p < 0.001. Good convergent validity between dimensions of AFEQT and of Life Attitude Profile-Revised in young and middle-aged participants showed correlations for superordinate indices between -0.23 and 0.72, and an overall p < 0.001. Dying people scored significantly higher for all dimensions, especially “altruistic preoccupation” and “reconciliation with existence” than people in other life stages (p < 0.01- < 0.001). Personality traits of “openness” and “agreeableness” are positively associated with higher scoring of AFEQT dimensions. About 77% of dying participants reported a personal benefit through the interview questions.

**Conclusions:**

With proximity to death, the anthropological dimensions proposed scored significant higher than in other stages of life, reflecting a stronger awareness, confrontation and reconciliation with the end of their own life. These dimensions, especially preoccupation for related persons and coexistence of acceptance and struggle with death have to be taken into account in a sensitive way by supporting dialogues with dying people and their relatives.

**Trial registration:**

Observational cross-sectional study.

**Supplementary Information:**

The online version contains supplementary material available at 10.1186/s12904-021-00818-y.

## Background

In addition to adequate medical and psychological care, spiritual care is of central importance in palliative medicine since it implies creative, narrative, and ritual work [1–4]. In its most general sense, spiritual care can be defined as supporting a terminally ill individual in the search for personal meaning in his or her actual situation with regard to the course of life and the significant relationships with other people [1, 4]. In a recent systematic narrative review of studies conducted in European countries, Gijsberts et al. [1] identified 53 articles covering a broad spectrum of research topics with regard to spiritual care. In the discussion of their results, the authors came to the conclusion that there is a particular need for the development of standardised outcome measures [1]. In their review of instruments measuring spirituality in clinical research, Monod et al. [5] mention the broad spectrum of theoretical conceptualisations of spirituality in clinical settings and the lack of instruments which assess the patients’ current state of spiritual state and their need for spiritual intervention [5]. In this article, a new theoretical approach towards a better comprehension of existential issues at the end of life was outlined on an anthropological basis assuming six dimensions that are activated by proximity to death. This approach displays a relevant interface with spiritual care approach since both of them transfer humanistic assumptions into real care considering spiritual needs of human beings in their confrontation with their own death.

### Philosophical framing

There are three ways of “ceasing to be”: for matter (inorganic world), this means “cessation” or a “decay of structures”, for living beings “perishing”, and for humans “dying” [6]. From an anthropological perspective, “death” means for humans not only “finiteness”, but rather “mortality” as the “continuous presence of death though its absence in life”, as proposed by Paul Landsberg ([7], p. 36). “To be mortal” means the unavoidable annihilation of individual existence and personal continuity. In the first-person perspective, death means experiencing an awareness of the inevitability of having to die and dying itself as “suffering death” ([8], p. 33). Dying means in the second person perspective “dying for someone”, for one *other* because humans are not only beings, but also beings-with-other-beings due to the fundamental structure of mutuality ([9], p. 100; 7, pp. 39–46; [10], p. 393, pp. 520–524; [11], pp. 147–152). From a third-person-perspective, death has to be considered as an ontic and not an ontological phenomenon meaning that dying is considered from a medical and biological perspective as agony and death, and the deceased person from an external perspective as a corpse.

Consciousness and its ability to symbolise is the condition for the possibility that humans know about themselves as subjects. Only humans have a concept of their selves as an identity over time that is named the “self” because of the recursiveness when the subject calls himself “me”. When a person is really aware about his unavoidable mortality, because of suffering from a mortal disease, it can be assumed that the consciousness triggers a biographical evaluation in the retrospective as well as anticipating personal circumstances around the own death in order to find a way to cope with the personal significance of ceasing-to-be for oneself and for relevant others. This process can be defined metaphorically as a “farewell to the own existence”. The authors assume that this anticipating effort runs along anthropological issues that are activated in a genuine way, facing the own unavoidable death due to a certain mortal disease. Anthropologically, “anticipation” means the assumption of existential and relevant actual events or scenarios in the future of every human being that have to be taken into account in the present in order to give the individual’s life a direction from now on, but in a very personal manner that cannot be subject of moral judgements about the right way of dying.

### The “Anticipated Farewell to Existence” theory

Based on the concept of *Daseinsverabschiedung* [12], a theory of “Anticipated Farewell to Existence” was introduced as a personal task in a panoptic examination of one’s own death, of the lived and unlived, as well as of the remaining lifetime on the basis of fundamental dimensions of human existence. The deeper sense of “farewell” was drawn up by Elisabeth Kübler-Ross as a personal leave-taking from life [13] and by Ralf Marten as the anticipation of death as farewell process facing her-/himself as well as facing survivors ([11], pp. 158–161). These grounding dimensions, based on a fundamental ability to symbolise, are extracted and refined from relevant works to this issue [14–16]: ipseity-selfhood, interpersonality, temporality, corporeality, worldliness, and transcendence [12]. These dimensions are generated from a theoretical approach and regarded as fundamental for human beings; thus, dimensions drawn up a priori cannot be further reduced, but yet deductively justified.

**Fig. 1:**
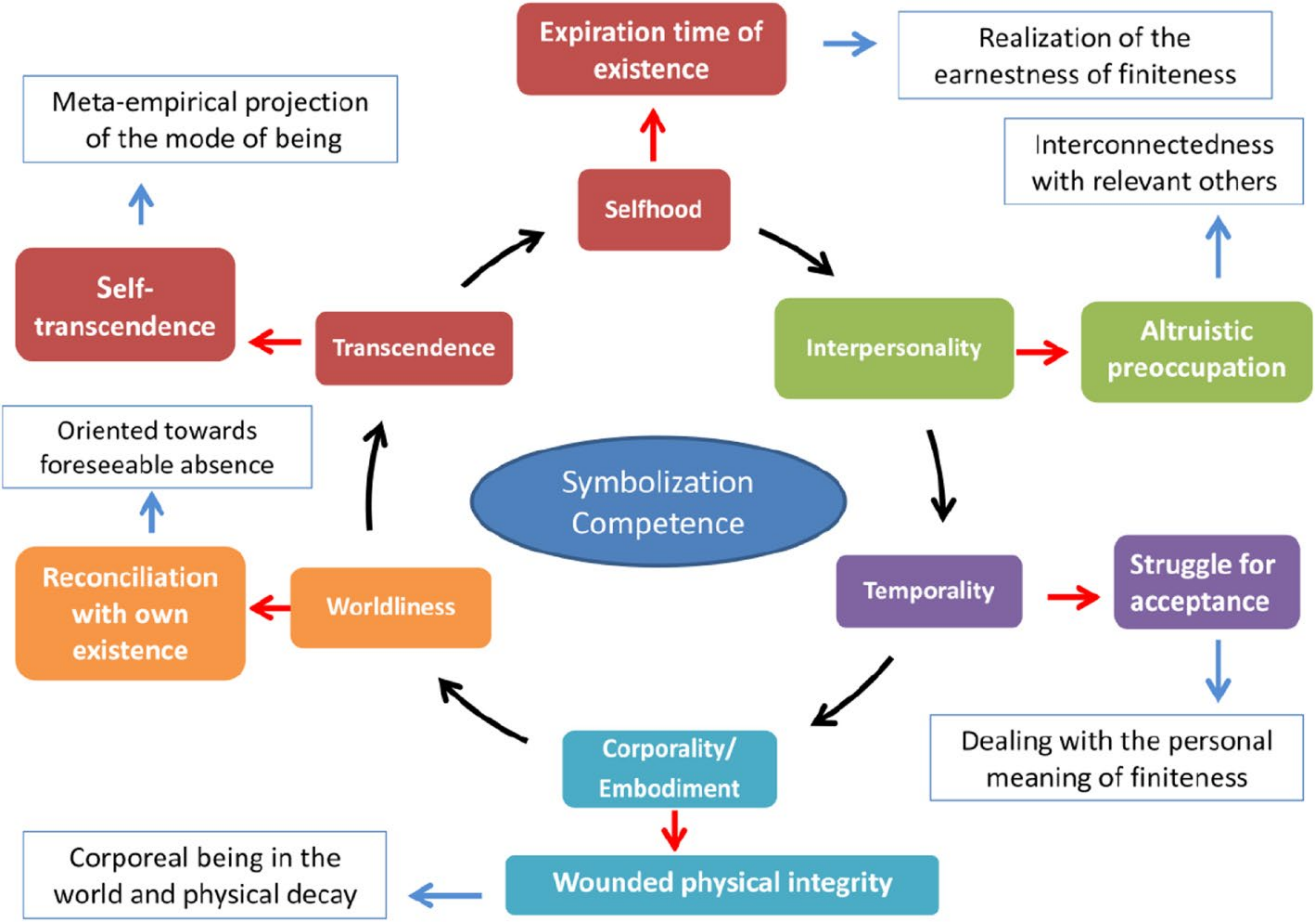
This scheme is based on the already proposed and justified fundamental dimensions. From each fundamental dimension, another dimension is derived in the confrontation with own death, which together configures the “Anticipatory Farewell to Existence” construct. This construct is rooted in the analytical structure of human existence. A phenomenological-anthropological scope is added to each of these dimensions

Each of these becomes transformed for the human phenomenon of the confrontation with death in “struggle for acceptance” (for “ipseity/identity”), “reconciliation with one’s own existence” (for “worldliness”), “wounded physical integrity” (for “corporeality”), “expiration of the time of existence” (for “temporality”), “altruistic preoccupation” (for “interpersonality”), and “self-transcendence” (for “ability to transcend”). These dimensions have already been defined and justified in depth [12]. The generalisation of the dimensions is systematically applied, as shown in Fig. 1. The six dimensions proposed and their respective complementary two-factor structures are presented in Supplementary Table 2 and are defined as follows:

The dimension of the “expiration of the time of existence” is aimed at the realisation that by its nature, one’s own existence comes to an unavoidable end. This awareness of one’s own cessation-to-be may initiate a process of self-distancing (“farewell”) and also acceptance (“conclusion”).

The dimension of “reconciliation with one’s own existence” aims for an emotional balance that attempts to bring the lived and the unlived together in a personal sense of coherence. This balance is not arithmetic. Rather, it reflects the degree of life realisation (“fulfilment of existence”) and the perceived extent of coherence (“harmony”).

The dimension of “struggle for acceptance” is not meant teleologically, but as an open process that expresses the real dissension and existential contradictions that arise as existence in the face of the inevitable own death. This state of foreseeable, inescapable cessation of being sets an ambivalence in motion in the deep layers of our existence that moves, to varying degrees, between an attitude of “acceptance” and “resistance”. These are the only factors within a dimension that are not complementary but opposite. “Resistance” means that despite the awareness of one’s own finiteness, an emotional reaction of defensiveness and reluctance arises when facing death. “Acceptance” means the degree of assumption of the unavoidable finiteness and the lived as well as the unlived life from an evaluative biographical retrospect.

From a medical-anthropological point of view, the dimension of “wounded physical integrity” means the inclusion of embodiment in this theoretical construct, because the body is in a state of irreversible decay and leads to dependence. This body-related condition is regarded as essential for examination of the dying process beyond pain and functional disorders. Two aspects are considered in this dimension: the biological body (“physical disability”) and the experienced body as interacting closely with helping people (“corporeality as a presence”).

The dimension of “altruistic preoccupation” means that the process of *Daseinsverabschiedung* (farewell to existence) implies the inclusion of the compassionate. Every self is existentially interwoven with others with whom there is a deeper emotional bond. Thus, farewell to existence must consider relevant others in a double sense: others as bearers of the trace of one’s own existence (“bequest”) and others as addressees of the efforts to relieve them emotionally through an ego-decentred attitude (“charity”).

The dimension “self-transcendence” means the reflective detachment from painful circumstances at the end of life in the certainty of one’s own death. If transcendence as self-distancing occurs, it will be gradual. The factor “permanence” means the striving for, or the disregard of, a spiritual or material memory by others who were possibly earlier addressees of one’s own working, loving, and living. The factor “metaphysical rise” means the conviction or rejection that one’s own existence in the world could possibly change into another way of being (not only in the religious sense) and thus the essence of being experiences a continuation.

## Methods

### Aim

The main objective of the study is to quantitatively compare the dimensions and factors of the *Daseinsverabschiedung* theory by means of the AFEQT questionnaire between dying people in palliative care and people in other stages of life. The second objective is to examine the dimensional structure and the psychometric properties (objectivity, reliability, convergent validity, and criterion validity) of the AFEQT questionnaire as a formative model. The criterion validity consists of the increasing importance of outlined anthropological dimensions that are especially activated when facing one’s own death, with proximity to death throughout life stages.

### Study design

This is a cross-sectional, analytical study performed using the novel assessment instrument AFEQT to measure differences in the degree of confrontation with one’s own death. The participants were recruited from several centres due to the nature of the subsamples (people in palliative care, older people in nursing homes, companies, associations, students) in the sense of a convenience sampling in order to achieve the subsamples we aim to compare. This questionnaire was first developed in German after a Delphi forum consisting of four professionals and according to the rules to phrase questions [17]. In a pilot phase, five healthy middle-aged participants completed the scale in order to improve the comprehensibility of sentences; dying people were not invited in the pilot phase in order to avoid unnecessary psychological strain. The German questionnaire was translated into English to be published internationally; a native speaker performed a reverse translation with 95% matching and correction of the few discrepancies (see Supplementary Table 1). The sample consists of a total of 485 participants, divided into the following partial samples: dying individuals cared for in palliative wards and in hospices (n = 121) consisting of people who know about their disease and prognosis and have a life expectancy of few weeks on average according to palliative doctor (post investigation records showed an average survival time about 9 weeks, mostly less than 7 weeks; only 1 participant reached 9 months); old people living in nursing homes not suffering from a (known) mortal disease (n = 62); young adults (18‒25 years old, n = 152); and middle-aged adults (40‒55 years old, n = 150). The subjects were consecutively included in the study if they had given their consent. All hospice residents and palliative patients were from the same city and were assessed by the same physician (a specialist in psychosomatics and experienced psycho-oncologist); the nursing home residents came from four different nursing homes, with half assessed by a psychologist and half by a physician; young adults (mostly students) and middle-aged adults came from the same state in Germany and were interviewed by two doctorate candidates. Inclusion criteria for the hospice and palliative patients were: suffering from a disease in the terminal stage; sufficient cognitive and/or verbal abilities to be able to deal with the questions ‒ the answers were entered by the interviewer in the case of severe weakness; informed consent to participate in the investigation. In nursing homes, an additional inclusion criterion was considered: not suffering from a known mortal illness. Inclusion criteria for young adults and middle-aged adults were informed consent, age and not suffering from a severe illness. The exclusion criteria resulted from the above-mentioned inclusion criteria. Four palliative/hospice patients required a second interview because of psychological strain due to their first interview. All participants from the hospice/palliative group were asked at the end of the interview to indicate on a scale of -4 to + 4 how intensively this interview had influenced them in their confrontation of existential issues.

The study, including all implemented instruments, was approved by the ethics committee of the University of Ulm (Germany) for the examination of dying people (registration no. 45/15), for the extension of the study to nursing homes (registration no. 235/18), and for the extension of the study to young and middle-aged adults (registration no. 02/19). All participants provided written informed consent.

### Description of sample

The four subsamples were compared using 17 socio-demographic, medical, and personality variables. Women were overrepresented, especially in the subsample of elderly people (on average 85.0 years old, 84% women). High-school graduates are overrepresented in the subsample of young adults (on average 20.5 years old, 72% high school). With regard to parenthood, the subsamples of middle-aged adults (on average 48.7 years old, 91% parents) and dying individuals (on average 70.0 years old, 83% parents) were overrepresented. Only 21% of elderly people lived in couples, but 91% of middle-aged and 49% of dying individuals did. An immigration background was reported by 14% of the participants. No differences were found for psychiatric hospitalisations or any psychiatric treatment. A quarter of elderly people (23%) and four out of five dying individuals received psychopharmaceuticals. Dying people reported much more psychological and physical stress as well as a more impaired performance status than old people living in nursing homes. There are few personality trait differences with the exception being, that dying people showed more agreeableness and older people more conscientiousness on average (see Table 1). These variables were considered as control variables in multivariate regression models.

**Table 1:** Multidimensional profile of compared groups. Differences are based on Mann–Whitney tests or variance analyses ‒ Scheffé test (metric variables) and chi-square test (categorical variables)

	**Whole sample** **(N = 485)**	Young adults (1) (N = 152)	Middle-aged (2) (N = 150)	Elderly people (3) (N = 62)	Dying persons (4) (N = 121)	**Differences**
	**M (SD) or %**	**M (SD) or %**	**M (SD) or %**	**M (SD) or %**	**M (SD) or %**	**P / effect size**
***Socio-demographic variables***						
**1. Age**	49.8 (23.9)	20.5 (2.4) **R:** 18‒25	48.7 (4.4) **R:** 40‒55	85 (7.1) **R:** 67‒97	70 (11.0) **R:** 40‒91	
**2. Gender** (% women)	66%	63%	67%	84%	61%	0.012 / 0.15
**3. Education** (% ≥ secondary)	43%	72%	51%	33%	51%	< 0.001 / 0.50
**4. Immigration background**	14%	17%	7%	12%	20%	0.023 / 0.14
**5. Parenthood**	57%	1.3%	91%	66%	83%	< 0.001 / 0.78
**6. Currently living in couple**	56%	41%	91%	21%	49%	< 0.001 / 0.50
***Medical history variables***						
**7. Psychiatric hospitalization (lifetime)**	7.4%	8.5%	5.4%	6.5%	8.9%	n.s
**8. Outpatient psych. treatment (lifetime)**	20%	20%	21%	13%	24%	n.s
**9. Current psychopharmaceuticals**	25%	6%	2%	23%	80%	< 0.001 / 0.76
***Additional clinical variables***						
**10. Σ psychological stress (PO-Bado)**				4.36 (4.54)	9.62 (7.41)	< 0.001 / 0.80
**11. Σ physical stress (PO-Bado)**				3.74 (3.08)	7.38 (4.06)	< 0.001 / 0.97
**12. ECOG**				0.97 (0.90)	3.13 (0.72)	< 0.001 / 2.75
***Personality dimensions***						
**13. BFI ‒** Neuroticism	2.86 (0.90)	2.71 (0.68)	3.02 (0.57)	2.93 (1.07)	2.82 (1.28)	2 > 1
**14. BFI ‒** Extraversion	3.12 (0.93)	3.14 (0.68)	3.26 (0.77)	3.00 (0.98)	2.99 (1.27)	Ø
**15. BFI ‒** Openness	3.28 (0.93)	3.18 (0.75)	3.14 (0.73)	3.42 (0.96)	3.52 (1.23)	4 > 1, 2
**16. BFI ‒** Agreeableness	3.51 (0.79)	3.40 (0.70)	3.45 (0.64)	3.42 (0.84)	3.78 (0.96)	4 > all
**17. BFI ‒ C**onscientiousness	3.38 (0.86)	3.07 (0.68)	3.06 (0.63)	4.25 (0.83)	3.72 (0.88)	4 > 1, 2; 3 > 1, 2, 4

**Σ = **sum of values of all items;** M** = mean; **SD** = standard deviation; **Effect size**: Cramer’s V for categorical variables, Cohen’s d for metric variables;** p** = level of significance of test; **BFI** = Big Five Inventory-10; **ECOG** = Eastern Cooperative Oncology Group; **PO-Bado** = Psycho-Oncology Basic Documentation; **R** = Age range

### Assessment instruments

#### Anticipated Farewell to Existence Questionnaire (AFEQT)

This questionnaire is based on an anthropological theory that was previously developed for anticipatory dealing with one’s own death [12, 18]. However, the dimension “wounded physical integrity” was not included in this investigation, as dying people who are physically decaying have, in principle, a clearer physical impairment than other groups that could be considered. The questionnaire consisted of 51 questions related to five dimensions and 10 factors. The individual values are averaged over each dimension and factor.

#### The Basic Documentation for Psycho-Oncology (PO-Bado)

From this validated basic documentation, the sections “Somatic Stress” (four items) and “Psychological Stress” (eight items) have been selected for the present study. Each item is answered on a Likert scale ranging from 0 (“not suffering”) to 4 (“suffering a lot”) [19]. The questions refer to the subjective experience of the patient and not to the intensity of the symptom [20]. In the study, the variable “sum score” is recorded as a simple addition of all values for each of the two scales.

#### Eastern Cooperative Oncology Group (ECOG)

It measures the current functional status on a scale from 0 (“normal activity”) to 4 (“patient is totally confined to bed or chair”) [21]. This index is also part of the PO-Bado described above [20].

#### Big Five Inventory (BFI-10)

This is a validated scale for the dimensional recording of five personality dimensions (neuroticism, openness, conscientiousness, extraversion, and agreeableness) with 10 items, two per scale, which are averaged (one item must always be recoded). The answers are given on a Likert scale ranging between 1 and 5 [22].

#### Life Attitude Profile-Revised (LAP-R)

Validated questionnaire consisting of 48 items distributed in six dimensions [23]: Life Purpose (LP) ‒ Orientation and life tasks, feeling of individual significance); Coherence (CO) ‒ Awareness and acceptance of oneself, with others, and with life); Choice/Responsibleness (CR) ‒ Sense of responsibility, decision-making, freedom, and control); Death Acceptance (DA) ‒ Death as part of life, absence of fear thereof); Existential Vacuum (EV) ‒ Negative scale: Lack of purpose, goals, direction, interests, and decision-making); Goal Seeking (GS) ‒ Search for challenges that could enrich life). As well as dimensions, there are two superordinate indices: Personal Meaning Index (PMI) as an addition of Life Purpose and Coherence (PMI = PU + CO) and Existential Transcendence (ET) as an addition of Life Purpose, Coherence, Choice/Responsibleness, and Death Acceptance less the addition of Existential Vacuum and Goal Seeking (ET = (LP + CO + CR + DA) – (EV + GS)) [24]. In this investigation, the German version by Mehnert et al. [25] was implemented.

### Statistics

All metric variables considered were tested for normal distribution using the Shapiro–Wilk test. If the significance level (p-value) was < 0.05, the assumption of a normal distribution was rejected. The 17 variables of the multidimensional profile were compared for four subsamples by means of variance analyses (differences were assessed with the Scheffé test, and for two subsamples (dying people and old people in nursing homes) by means of the non-parametric Mann–Whitney U test.

Differences between dying participants reporting a positive influence by interview on confrontation with existential issues and reporting a neutral/negative influence were assessed for all dimensions and factors by means of the Mann–Whitney U-test and effect sizes of differences.

For each dimension and corresponding factors, the four subsamples were compared graphically by means of Whisker boxes and statistically by means of variance analyses. The relationship between the dimensions and sub-dimensions for the compared four subsamples was investigated with correlation matrices (product-moment correlations or Pearson correlations), stating the correlation coefficient |r| and the significance level (p). We consider values of |r| between 0.10 and 0.30 as a low correlation, between 0.3 and 0.5 as a medium correlation, and > 0.5 as a high correlation.

As for psychometric properties, objectivity, reliability, and validity were explored. Objectivity includes the homogeneity of the conditions of the investigation: all old and dying participants were accompanied when answering the questionnaires and were not left alone with their answers, whereby mainly supporting and clarifying statements were made; more sprightly participants were more independent and weakened patients were dependent on support. Old people living in nursing homes required support, but less so than dying people because they were not as weak. Young and middle-aged adults did not need further support. In this respect, the objectivity of implementation was not completely homogeneous for naturalistic reasons. The objectivity of evaluation was more homogeneous, but in the case of dying people, the answers sometimes required confirmation, clarification, or interpretation of the statement in light of the underlying construct.

The reliability (in the sense of internal consistency) of AFEQT was tested using Cronbach’s alpha. Two parameters were assessed for each item and for the overall test, allowing comparisons between the whole (test) and the elements (items): a) average inter-item correlation (AIC): this is the correlation of the questions with each other; if the correlation is too low, there is little homogeneity of the questionnaire or the dimension studied; if it is too high, the questions are redundant; it is assumed that values ≥ 0.30 indicate a good correlation between items; b) alpha: this is a measure of the internal consistency of a test, i.e. how strongly the questions of a scale are related to each other; it is assumed that a value of ≥ 0.70 for the entire scale indicates good internal consistency; this overall value improves if the individual items with an alpha of the respective item greater than the alpha of the overall test are removed from the model. Validity was explored by means of two approaches: *convergent validity* was assessed with the Life Attitude Questionnaire-Revised (LAP-R), as it also deals with existential issues, and even confronts mortality; the reservation has to be made that only young and middle-aged adults (N = 302) completed this questionnaire due to the observed overstraining of nursing home, palliative care, and hospice participants, so we decided against assessing old people with a supplementary questionnaire.

The criterion validity arises from the underlying anthropological model suggesting that the importance of the examination of one's own existence grows with the closeness of the individual to his own death. Therefore, we expect that the levels of awareness, confrontation and also acceptance of the finitude of life increases with the participants’ knowledge of the proximity to their own death, independent of the individuals' age. The differences in scoring dimensions and factors in the course of life were examined in a first step by means of bivariate variance analyses, in a second step by means of multivariate linear regression analyses using robust estimators, and in a third step by means of multivariate regression models using propensity scores. Bivariate tests were performed by means of variance analyses and a post hoc Scheffé test. Multivariate regression analyses consider dimensions and factors of AFEQT as dependent and subsamples as independent group variables (dying people as base outcome) as well as 13 additional control variables; robust standard errors are used to obtain unbiased standard errors of coefficients under heteroscedasticity. Post hoc power (1-β) was calculated for these models. Propensity scores (for each subsample) compress all 13 control variables; F-statistics improves the application of propensity scores because there are more degrees of freedom.

All statistical calculations were performed with the statistical package StataMP 13.0 and G*Power 3.1.

## Results

**Fig. 2:**
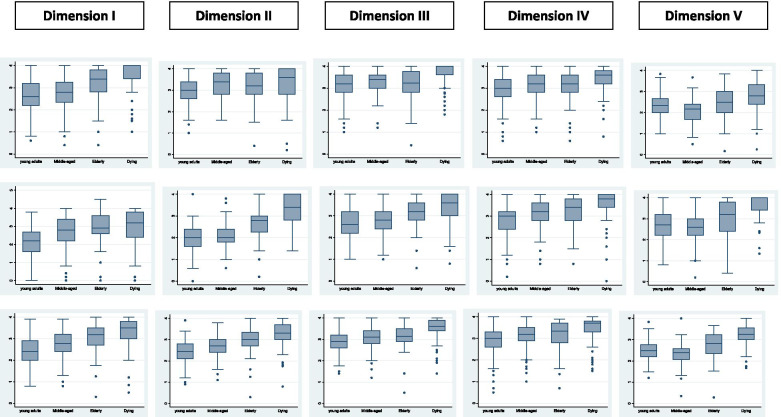
Whisker box comparing medians and scattering of all factors and dimensions of AFEQT among compared subsamples. Structure of the box: the box is delimited by the 1st quartile (25%) and 3rd quartile (75%); the median is the horizontal line in the box; the antennas (whiskers) correspond to the minimum and maximum data: points outside (*) are outliers if 1.5 times the interquartile distance (Q1‒Q3) is exceeded upwards or downwards or extreme outliers if > 3 times the interquartile distance

The raw data were obtained from three prior field surveys [18, 26, 27], but data are merged and combined for subsample comparisons with a new criterion validity for the whole sample. The Shapiro–Wilk test demonstrates that all dimensions are non-normally distributed for the whole sample (N = 485), with the exception of “resistance”. In contrast, most of the dimensions are normally distributed for the subsample of young adults. The number of normally distributed dimensions decreases with age; for dying people, there is a very positive skew, indicating that the tail is on the right side of the distribution, as illustrated by the differences between the mean and median (see Table 2). The higher the age, the higher the scores of all dimensions and factors of AFEQT, especially for dying people (see Fig. 2 and Table 2); this trend is also evident if one makes comparisons on a question by question basis (see Supplementary Table 3). The dispersion of dimension and factor scores measured by variation coefficients is low to moderate (13‒32%), especially for the dimension “altruistic preoccupation”. Tendentiously, the lowest values for variation coefficients are found for dying people, but this group displays more outliers (see Fig. 2 and Table 2). In the palliative/hospice subgroup, 77.1% of participants reported a positive and very positive influence of the contents of interview on their confrontation with existential issues at the end of life, 20.3% reported a neutral influence and only 2.6% reported a negative influence. By means of bivariate analyses, between positive and neutral/negative appraisal, there were only significant differences for the dimensions altruistic preoccupation (p = 0.05, Effect size = 0.54) and struggle for acceptance (p = 0.05; Effect size = 0.30).

**Table 2:** Comparison of means among explored subsamples by variance analyses (Scheffé test)

	**Young adults (1)** (N = 152)			**Middle-aged (2)** (N = 150)			**Elderly people (3)** (N = 62)			**Dying persons (4)** (N = 121)			**Differences** (Variance analyses, Scheffé test)		
	**M (SD)**	**Vc**	**Md**	**M (SD)**	**Vc**	**Md**	**M (SD)**	**Vc**	**Md**	**M (SD)**	**Vc**	**Md**	**Bartlett**^**p**^	**F **^**p**^	**Significant diff**
a) Permanence	2.62 (0.78)	30%	2.6	2.75 (0.76)	28%	2.8	3.16 (0.86)	27%	3.4	3.60 (0.63)	17%	4.0	9.33*	49.4***	3 > 1, 2; 4 > 1, 2, 3
b) Metaphysical rise	2.21 (0.69)	31%	2.2	2.74 (0.82)	30%	2.8	2.94 (0.87)	30%	2.9	3.09 (0.84)	27%	3.2	8.42*	29.7***	3 > 1; 4 > 1, 2
**I: Self-transcendence**	2.42 (0.62)	26%	2.4	2.74 (0.61)	22%	2.8	3.05 (0.65)	21%	3.2	3.25 (0.65)	20%	3.5	0.56 ^n.s^	53.2***	2 > 1; 3 > 1, 2; 4 > 1, 2, 3
a) Conclusion	2.97 (0.64)	22%	3.0	3.26 (0.58)	18%	3.4	3.20 (0.74)	23%	3.2	3.32 (0.78)	23%	3.6	14.1*	7.5***	2 > 1; 4 > 1
b) Farewell	1.89 (0.59)	31%	2.0	2.09 (0.52)	25%	2.0	2.67 (0.69)	26%	2.8	3.24 (0.69)	21%	3.4	12.5**	128***	2 > 1; 3 > 1, 2; 4 > 1, 2, 3
**II: Expiration time of own existence**	2.43 (0.53)	22%	2.5	2.67 (0.48)	18%	2.7	2.93 (0.63)	22%	3.0	3.28 (0.55)	17%	3.3	7.1 ^n.s^	60.7***	2 > 1; 3 > 1,2; 4 > 1, 2, 3
a) Bequest	3.12 (0.62)	20%	3.2	3.29 (0.49)	15%	3.4	3.14 (0.71)	23%	3.2	3.72 (0.48)	13%	4.0	20.9***	29.0***	4 > 1, 2, 3
b) Charity	2.64 (0.65)	25%	2.6	2.82 (0.56)	20%	2.8	3.12 (0.67)	21%	3.2	3.34 (0.69)	21%	3.6	6.7 ^n.s^	30.3***	3 > 1, 2; 4 > 1, 2
**III: Altruistic preoccupation**	2.88 (0.52)	18%	2.9	3.05 (0.46)	15%	3.1	3.13 (0.58)	18%	3.1	3.53 (0.47)	13%	3.6	5.6 ^n.s^	39.7***	2 > 1; 3 > 1; 4 > 1, 2, 3
a) Fulfilment	2.95 (0.71)	25%	3.0	3.15 (0.58)	18%	3.2	3.10 (0.75)	24%	3.2	3.41 (0.59)	13%	3.6	10.7*	11.8***	4 > 1, 2, 3
b) Harmony	2.79 (0.74)	26%	3.0	3.12 (0.62)	20%	3.2	3.21 (0.73)	23%	3.4	3.56 (0.67)	19%	3.8	4.9 ^n.s^	28.2***	2 > 1; 3 > 1; 4 > 1, 2, 3
**IV: Reconciliation with existence**	2.87 (0.68)	24%	3.0	3.13 (0.56)	18%	3.2	3.15 (0.68)	20%	3.3	3.48 (0.55)	15%	3.7	9.7*	22.6***	2 > 1; 3 > 2; 4 > 1, 2, 3
a) Resistance	2.36 (0.53)	22%	2.3	2.07 (0.53)	26%	2.2	2.45 (0.71)	29%	2.5	2.75 (0.68)	25%	2.8	16.1**	29.1***	1 > 2; 3 > 2; 4 > 1, 2, 3
b) Acceptance	2.64 (0.65)	25%	2.7	2.57 (0.64)	25%	2.6	3.00 (0.93)	31%	3.2	3.36 (0.50)	15%	4.0	34.9***	75.7***	3 > 1, 2; 4 > 1, 2, 3
**V: Struggle for death acceptance**	2.50 (0.43)	17%	2.5	2.34 (0.46)	20%	2.4	2.73 (0.64)	23%	2.8	3.21 (0.45)	14%	3.3	17.2**	82.6***	1 > 2; 3 > 2; 4 > 1, 2, 3

**Post hoc power (1-β) when effect size f**^**2**^ **= 0.25, α = 0.05, and critical F = 2.61 is 0.99**

**N** = sample size;** M** = mean; **SD** = standard deviation; **Vc** = Variation coefficient (SD/M) × 100; **Md** = median (50th percentile); **p** = level of significance: * < 0.05; ** < 0.01; *** < 0.001; **F** = F-statistics for the whole model; **significant diff.** = differences in Scheffé test among the four compared groups are statistically significant

Product-moment correlations between all dimensions for the four subsamples presented in Table 3 indicate a high correlation (mostly > 0.80) between dimensions for all subsamples. The correlations between dimensions mostly range between 0.30 and 0.50, but sometimes higher, especially for dimensions but less so for factors (see Table 3). An exception is the factor “resistance”, which hardly displays significant correlation (and with dimensions and factors of “reconciliation with own existence” even negative correlations); especially remarkable is the lack of association between “resistance” and “acceptance” for all subsamples (see Table 3).

**Table 3:** Correlation matrix for dimensions and factors for compared four subsamples

	**Self-transcendence**			**Expiration time of own existence**			**Altruistic preoccupation**			**Reconciliation existence**			**Struggle for acceptance**		
	**Factor Ia**	**Factor Ib**	**Dimension** **I**	**Factor IIa**	**Factor IIb**	**Dimension** **II**	**Factor** **IIIa**	**Factor IIIb**	**Dimension** **III**	**Factor IVa**	**Factor IVb**	**Dimension IV**	**Factor Va**	**Factor Vb**	**Dimension** **V**
	**r(1)/r(2)** **r(3)/r(4)**	**r(1)/r(2)** **r(3)/r(4)**	**r(1)/r(2)** **r(3)/r(4)**	**r(1)/r(2)** **r(3)/r(4)**	**r(1)/r(2)** **r(3)/r(4)**	**r(1)/r(2)** **r(3)/r(4)**	**r(1)/r(2)** **r(3)/r(4)**	**r(1)/r(2)** **r(3)/r(4)**	**r(1)/r(2)** **r(3)/r(4)**	**r(1)/r(2)** **r(3)/r(4)**	**r(1)/r(2)** **r(3)/r(4)**	**r(1)/r(2)** **r(3)/r(4)**	**r(1)/r(2)** **r(3)/r(4)**	**r(1)/r(2)** **r(3)/r(4)**	**r(1)/r(2)** **r(3)/r(4)**
Ia Permanence															
Ib Metaphysical rise	0.41/ 0.19 n.s. / 0.53														
**I Self-transcendence**	**0.86 / 0.73** **0.74 / 0.83**	**0.82 / 0.79** **0.78 / 0.91**													
IIa Conclusion	0.39 / 0.17 0.44 / 0.35	0.41 / 0.46 0.52 / 0.40	0.48 / 0.41 0.63 / 0.43												
IIb Farewell	0.35 / 0.25 0.34 / 0.34	0.36 / 0.43 0.35 / 0.30	0.42 / 0.45 0.45 / 0.36	0.47 / 0.51 0.55 / n.s											
**II Expiration time of own existence**	0.43 / 0.24 0.45 / 0.46	0.45 / 0.51 0.50 / 0.47	0.52 / 0.50 0.62 / 0.52	**0.87 / 0.88** **0.89 / 0.79**	**0.85 / 0.86** **0.87 / 0.71**										
IIIa Bequest	0.70 / 0.69 0.57 / 0.56	0.34 / 0.31 n.s. / 0.26	0.63 / 0.63 0.46 / 0.44	0.46 / 0.40 0.36 / 0.35	0.30 / 0.42 0.42 / 0.21	0.44 / 0.47 0.44 / 0.38									
IIIb Charity	0.37 0.38 0.26 / 0.27	0.32 / 0.39 0.29 / n.s	0.41 / 0.49 0.36 / 0.19	0.33 / 0.40 0.48 / 0.20	0.27 / 0.42 0.48 / 0.34	0.36 / 0.47 0.55 / 0.35	0.34 / 0.53 0.39 / 0.30								
**III Altruistic preoccupation**	0.65 / 0.60 0.50 / 0.48	0.41 / 0.40 0.25 / 0.19	0.64 / 0.63 0.49 / 0.36	0.48 / 0.46 0.50 / 0.32	0.35 / 0.48 0.54 / 0.35	0.49 / 0.54 0.59 / 0.45	**0.81 / 0.86** **0.85 / 0.72**	**0.83 / 0.89** **0.82 / 0.88**							
IVa Fulfilment	0.69 / 0.46 0.34 / 0.27	0.29 / 0.31 0.34 / 0.31	0.56 / 0.48 0.44 / 0.33	0.49 / 0.53 0.54 / 0.25	0.43 / 0.44 0.50 / 0.18	0.54 / 0.56 0.59 / 0.30	0.73 / 0.61 0.44 / 0.31	0.26 / 0.40 0.51 / n.s	0.59 / 0.57 0.57 / 0.26						
IVb Harmony	0.49 / 0.28 0.32 / 0.49	0.36 / 0.34 0.28 / 0.38	0.51 / 0.39 0.39 / 0.48	0.53 / 0.66 0.39 / 0.28	0.47 / 0.53 0.57 / 0.25	0.58 / 0.69 0.54 / 0.35	0.62 / 0.49 0.53 / 0.35	0.27 / 0.40 0.54 / 0.24	0.54 / 0.51 0.64 / 0.35	0.77 / 0.75 0.72 / 0.52					
**IV Reconciliation** **with own existence**	0.60 / 0.39 0.36 / 0.44	0.35 / 0.35 0.32 / 0.40	0.57 / 0.46 0.45 / 0.47	0.54 / 0.64 0.50 / 0.31	0.48 / 0.52 0.57 / 0.28	0.60 / 0.67 0.61 / 0.37	0.71 / 0.58 0.51 / 0.38	0.28 / 0.43 0.56 / 0.23	0.60 / 0.57 0.64 / 0.35	**0.94 / 0.93** **0.92 / 0.85**	**0.94 / 0.94** **0.92 / 0.89**				
Va Resistance	n.s. / n.s 0.37 / 0.23	n.s. / 0.25 n.s. / n.s	n.s./ 0.20 0.26 / n.s	n.s. / n.s 0.30 / n.s	n.s. / n.s 0.40 / 0.27	n.s. / n.s 0.40 / n.s	n.s. / n.s 0.32 / 0.20	n.s. / n.s 0.31 / n.s	n.s. / n.s 0.38 / 0.21	-0.17/ -.17 n.s. / n.s	-0.19/ -.22 0.36 / n.s	-0.19/ -.21 0.28 / n.s			
Vb Acceptance	0.36 / 0.16 n.s. / 0.25	0.30 / 0.47 n.s. / 0.28	0.40 / 0.42 n.s. / 0.31	0.38 / 0.56 0.29 / 0.27	0.38 / 0.42 0.30 / 0.37	0.44 / 0.56 0.33 / 0.42	0.33 / 0.37 n.s. / 0.20	0.26 / 0.42 0.39 / 0.18	0.36 / 0.45 0.34 / 0.23	0.40 / 0.34 0.41 / 0.27	0.41 / 0.43 0.57 / 0.27	0.43 / 0.41 0.53 / 0.31	n.s. / n.s n.s. / n.s		
**V Struggle for acceptance**	0.24 / n.s 0.31 / 0.31	0.23 / 0.47 n.s. / n.s	0.28 / 0.40 0.27 / 0.25	0.24 / 0.38 0.37 / n.s	0.25 / 0.34 0.44 / 0.41	0.28 / 0.41 0.46 / 0.31	0.16 / 0.23 0.31 / 0.26	0.26 / 0.30 0.46 / 0.21	0.26 / 0.31 0.46 / 0.28	0.19 / n.s 0.37 / 0.22	0.18 / 0.17 0.61 / n.s	0.20 / 0.16 0.54 / 0.22	**0.67 /0.66** **0.70 /0.84**	**0.79 / 0.74** **0.84 / 0.67**	

**R** = Pearson correlation coefficient; **(1)** = subsample young adults (18‒25); **(2**) = subsample middle-aged adults (40‒55); **(3)** = elderly in nursing homes; **(4)** = dying people; **n.s.** = not statistically significant; **statistical significance**: n.s. > 0.05; underlined, i.e. 0.16 means *p* < 0.05; not underlined means *p* < 0.005

Reliability was investigated considering internal consistency (Cronbach’s alpha). For the whole sample and for the subsamples of young adults, middle-aged adults, and elderly people, almost all dimensions showed an alpha > 0.70, whereas the different factors showed a greater heterogeneity. Only for dying people was alpha insufficient at the dimensional level (but sufficient for the whole scale), with the exception of the dimension “reconciliation with own existence” (see Table 4).

**Table 4:** Reliability of the “Anticipatory Farewell to Existence Questionnaire” along dimensions and each corresponding factor by means of Cronbach’s alpha applied to the four compared subsamples

	Whole sample (N = 485)		Young adults (N = 152)		Middle-aged adults (N = 150)		Elderly people (N = 62)		Dying persons (N = 121)	
	Cronbach’s α		Cronbach’s α		Cronbach’s α		Cronbach’s α		Cronbach’s α	
	**AIC**	**α**	**AIC**	**α**	**AIC**	**α**	**AIC**	**α**	**AIC**	**α**
a) Permanence	0.52	0.74	0.54	0.70	0.55	0.73	0.51	0.64	0.31	0.63
b) Metaphysical rise	0.38	0.56	0.21	0.47	0.78	0,69	0.57	0.62	0.37	0.44
**Dimension I: Self-transcendence**	***0.36***	***0.74***	***0.31***	***0.71***	***0.24***	***0.68***	***0.19***	***0.63***	***0.27***	***0.65***
a) Conclusion	0.50	0.66	0.19	0.48	0.43	0.70	0.30	0.56	0.53	0.60
b) Farewell	0.43	0.65	0.15	0.41	0.15	0.39	0.38	0.64	0.57	0.54
**Dimension II: Expiration time of own existence**	***0.38***	***0.74***	***0.33***	***0.71***	***0.22***	***0.72***	***0.26***	***0.71***	***0.15***	***0.51***
a) Bequest	0.25	0.68	0.29	0.72	0.23	0.64	0.31	0.62	0.34	0.61
b) Charity	0.24	0.52	0.16	0.39	0.21	0.51	0.30	0.53	0.39	0.53
**Dimension III: Altruistic preoccupation**	***0.22***	***0.72***	***0.24***	***0.72***	***0.16***	***0.72***	***0.27***	***0.71***	***0.22***	***0.63***
a) Fulfilment	0.29	0.64	0.35	0.70	0.24	0.70	0.48	0.68	0.27	0.52
b) Harmony	0.39	0.74	0.41	0.70	0.30	0.77	0.36	0.63	0.23	0.61
**Dimension IV: Reconciliation with own existence**	***0.35***	***0.83***	***0.43***	***0.85***	***0.27***	***0.85***	***0.40***	***0.80***	***0.27***	***0.73***
a) Resistance	0.58	0.58	0.20	0.53	0.45	0.70	0.41	0.58	0.48	0.47
b) Acceptance	0.50	0.66	0.20	0.48	0.28	0.60	0.88	0.80	0.15	0.31
**Dimension V: Struggle for acceptance of own death**	***0.30***	***0.70***	***0.18***	***0.63***	***0.24***	***0.71***	***0.43***	***0.75***	***0.07***	***0.42***
***Whole test***	0.23	0.91	0.18	0.90	0.16	0.91	0.22	0.90	0.11	0.83

**AIC** = average inter-item correlation; **α** = scale reliability coefficient; the reliability is defined as the square of the correlation between the measured scale and the underlying factor. When α of an item > α of the scale, then the item was removed

Convergent validity was assessed by means of a similar approach to the Life Attitude Questionnaire (LAP-R) which was used for the subsamples of young and middle-aged adults. The six dimensions of LAP-R and dimensions I‒IV of AFEQT were considered. As expected, the dimension “Existential vacuum” (LAP-R) was negatively associated with all dimensions of AFEQT (range of r = -0.37 to -0.60), except for “Resistance” (r = 0.45), which is similar to “Goal seeking” (LAP-R). Conversely, “Resistance” (AFEQT) was negatively associated with most dimensions and both superordinate indices, except for “Existential vacuum” (0.45) and “Goal seeking” (r = 0.27). The dimensions of LAP-R “Life purpose” and “Coherence” and the indices PMI and ET showed moderate to high associations (r = 0.26 to 0.72) with AFEQT dimensions I‒IV and Vb (acceptance), indicating a good convergence validity (see Table 5).

**Table 5:** Correlation matrix of the dimensions of LAP-R with dimensions of AFEQT (subsamples of young adults and middle-aged adults, n = 302)

	**I**	**II**	**III**	**IV**	**Va**	**Vb**
	**r **^**p**^	**r **^**p**^	**r **^**p**^	**r **^**p**^	**r **^**p**^	**r **^**p**^
Life purpose (PU)	0.42***	0.46***	0.51***	0.72***	-0.25***	0.26***
Coherence (CO)	0.56***	0.54***	0.48***	0.61***	-0.19**	0.36***
Choice/Responsibleness (CR)	n.s	n.s	0.22***	0.31	n.s	0.15**
Death acceptance (DA)	0.14*	0.34***	0.25***	0.14*	n.s	0.32***
Existential vacuum (EV)	-0.37***	-0.41***	-0.38***	-0.60***	0.45***	n.s
Goal seeking (GS)	n.s	-0.16**	n.s	-0.19***	0.27***	0.14**
Personal Meaning Index (PMI)	0.54***	0.55***	0.54***	0.72***	-0.23***	0.34***
Existential transcendence (ET)	0.45***	0.56***	0.51***	0.71***	-0.31***	0.30***

***Note*** : Alpha correction for 40 tests gives a significance value of p* < *0.0013*

Post hoc* power (1-β)when n* = *302, ρH1* = *0.30, α* = *0.05 amounts 0.99*

**r** = product-moment correlation coefficient; **p** = significance level of tests; n.s. = statistically not significant at 0.05‒level; * < 0.05; ** < 0.01; *** < 0.001; I = Self-transcendence; II = Expiration of one’s existence time; III = Altruistic preoccupation; IV = Reconciliation with own existence; Va = Resistance; Vb = Acceptance

The results of the multivariate regression analyses indicate that dying people scored significantly higher in all dimensions than young adults and middle-aged adults, and even higher than old people, with the exception of Self-transcendence (see Table 6). These results were confirmed by using propensity scores (see Table 7). The models also demonstrate an independent association of some regressors with dimensions of AFEQT: Parenthood is positively associated with “Self-transcendence” and “altruistic preoccupation” (similarly to women), “reconciliation with own existence” with women and higher education; with regard to personality traits, neuroticism is tendentiously negatively, whereas openness and greeableness are positively associated with assessed dimensions of AFEQT (see Table 6).

**Table 6:** Multivariate linear regression model dimensions and factors of “Anticipatory Farewell to Existence” questionnaire using robust standard errors

	**Self-transcendence**			**Expiration time of own existence**			**Altruistic preoccupation**			**Reconciliation existence**			**Struggle for acceptance**		
	**Dimension** **I**	**Factor Ia**	**Factor Ib**	**Dimension** **II**	**Factor IIa**	**Factor IIb**	**Dimension** **III**	**Factor** **IIIa**	**Factor IIIb**	**Dimension IV**	**Factor IVa**	**Factor IVb**	**Dimension V**	**Factor Va**	**Factor Vb**
	**Coef **^**p**^	**Coef **^**p**^	**Coef **^**p**^	**Coef **^**p**^	**Coef **^**p**^	**Coef **^**p**^	**Coef **^**p**^	**Coef **^**p**^	**Coef **^**p**^	**Coef **^**p**^	**Coef **^**p**^	**Coef **^**p**^	**Coef **^**p**^	**Coef **^**p**^	**Coef **^**p**^
**Base outcome: Dying people**															
1. Young adults	-0.73***	-0.64***	-0.83***	-0.77***	-0.39*	-1.14***	-0.56***	-0.51***	-0.61***	-0.68***	-0.61***	-0.74***	-0.54***	n.s	-0.92***
2. Middle-aged	-0.75***	-1.02***	-0.46**	-0.62***	n.s	-0.99***	-0.63**	-0.74***	-0.51***	-0.53***	-0.49***	-0.57***	-0.81***	-0.49***	-1.17***
3. Elderly people	n.s	n.s	n.s	-0.48**	n.s	-0.71***	-0.48**	-0.58***	-0.37*	-0.62***	-0.62**	-0.61***	-0.81***	n.s	-1.28***
Gender (1 = man)	n.s	n.s	n.s	n.s	n.s	n.s	-0.10*	-0.16**	n.s	-0.18**	n.s	-0.23***	n.s	n.s	n.s
Education	n.s	n.s	n.s	n.s	n.s	n.s	n.s	0.13*	n.s	0.16*	0.16*	0.15*	-0.15**	-0.16***	n.s
Immigration history	n.s	n.s	n.s	n.s	n.s	n.s	n.s	n.s	n.s	n.s	n.s	n.s	n.s	n.s	n.s
Parenthood	0.28*	0.45**	n.s	n.s	n.s	n.s	0.22*	0.37**	n.s	n.s	n.s	n.s	n.s	n.s	n.s
Partnership	n.s	n.s	n.s	n.s	n.s	n.s	n.s	0.16**	n.s	n.s	n.s	n.s	n.s	n.s	n.s
Psych. hospitalization	n.s	n.s	n.s	n.s	n.s	n.s	n.s	n.s	n.s	n.s	n.s	n.s	n.s	n.s	n.s
Psych. treatment	n.s	n.s	n.s	n.s	n.s	n.s	n.s	n.s	n.s	n.s	n.s	n.s	n.s	n.s	n.s
Psychopharmaceuticals	n.s	n.s	n.s	n.s	n.s	n.s	n.s	-0.24**	-0.08*	n.s	n.s	n.s	n.s	n.s	n.s
BFI ‒ Neuroticism	n.s	n.s	n.s	n.s	-0.08*	n.s	n.s	n.s	n.s	-0.11***	-0.09**	-0.12***	-0.06*	n.s	-0.08*
BFI ‒ Extraversion	n.s	n.s	n.s	n.s	n.s	n.s	n.s	0.06**	n.s	n.s	n.s	n.s	n.s	n.s	n.s
BFI ‒ Openness	0.11**	0.12***	0.11*	0.70*	n.s	0.12**	0.08**	0.06**	0.11**	0.08*	n.s	0.10**	0.08**	0.08*	0.08*
BFI ‒ Agreeableness	0.18***	0.26***	0.10*	0.90**	n.s	0.10*	0.19***	0.18***	0.20***	0.20***	0.21***	0.20***	n.s	n.s	n.s
**BFI-**Conscientiousness	n.s	n.s	n.s	n.s	n.s	-0.10*	n.s	n.s	n.s	-0.11*	n.s	-0.12**	n.s	n.s	n.s
***N***	428	428	428	428	428	428	428	428	428	428	428	428	428	428	428
***F***^***prob>F***^	16.8***	21.5***	8.3***	13.0***	3.1***	27.8***	15.6***	13.4***	8.5***	12.5***	7.4***	14.1***	22.6***	7.1***	25.0***
***R***^***2***^	0.33	0.37	0.19	0.33	0.10	0.50	0.36	0.33	0.25	0.28	0.21	0.29	0.41	0.20	0.40

**Post hoc power (1-β) by effect size f**^**2**^** = 0.15, α = 0.05, and critical F = 1.67 is 1.00**

**Gender:** 0 = women; 1 = men; **Parenthood, partnership, immigration, psychiatric hospitalization lifetime, psychiatric treatment lifetime, current intake of psychopharmaceuticals:** 0 = no, 1 = yes; **BFI** = Big Five Inventory-10; **Coef**. = Robust regression coefficient; **p** = level of significance of test; **N** = size of sample; **F** = statistic of model; **R**^**2**^ = explained variance of dependent variable; **p** = * < 0.050; ** < 0.01.; *** < 0.001; **Ia** = Permanence; **Ib** = Metaphysical rise; **IIa** = Conclusion; **IIb** = Farewell; **IIIa** = Bequest; **IIIb** = Charity; **IVa** = Fulfilment; **IVb** = Harmony; **Va** = Resistence; **Vb** = Acceptance

**Table 7:** Multivariate regression analyses for dimensions and factors of “Anticipatory Farewell to Existence” questionnaire using propensity scores

	**I: Self-transcendence**			**Permanence**			**Metaphysical rise**		
	**Coeff**	**t**	**p**	**Coeff**	**t**	**p**	**Coeff**	**t**	**p**
*Base outcome: Dying people*									
ps1 (young adults)	-0.97	-9.84	< 0.001	-1.08	-10.4	< 0.001	-0.87	-7.27	< 0.001
ps2 (middle-aged)	-.062	-6.17	< 0.001	-0.86	-8.46	< 0.001	-0.36	-2.78	0.006
ps3 (elderly)	-0.64	-1.27	n.s	-0.48	-1.62	n.s	-0.20	-0.63	n.s
N / F / prob > F / R^2^	428 / 37.3 / < 0.001 /0.22			428 / 44.4 / < 0.001 /0.21			428 / 22.0 / < 0.001 /0.13		
	**II: Expiration existence**			**Conclusion**			**Farewell**		
*Base outcome: Dying people*									
ps1 (young adults)	-0.88	-10.53	< 0.001	-0.32	-2.92	< 0.001	-1.44	-14.73	< 0.001
ps2 (middle-aged)	-0.62	-7.49	< 0.001	0.02	0.17	n.s	-1.27	-12.88	< 0.001
ps3 (elderly)	-0.70	-2.82	0.005	-0.34	-1.10	n.s	-1.06	-3.54	< 0.001
N / F / prob > F / R^2^	428 / 38.0 / < 0.001 / 0.24			428 / 6.6 / < 0.001 / 0.04			428 / 80.3 / < 0.001 /0.40		
	**III: Altruistic preoccupation**			**Bequest**			**Charity**		
*Base outcome: Dying people*									
ps1 (young adults)	-0.69	-9.05	< 0.001	-0.62	-7.30	< 0.001	-0.76	-7.89	< 0.001
ps2 (middle-aged)	-0.45	-6.02	< 0.001	-0.35	-4.46	< 0.001	-0.54	-5.76	< 0.001
ps3 (elderly)	-0.67	-2.93	0.004	-0.76	-2.79	< 0.006	-0.58	-1.95	0.052
N / F / prob > F / R^2^	428 / 27.5 / < 0.001 / 0.18			428 / 17.8 / < 0.001 /0.12			428 / 21.5 / < 0.001/ 0.15		
	**IV: Reconciliation existence**			**Fulfilment**			**Harmony**		
*Base outcome: Dying people*									
ps1 (young adults)	-0.61	-6.81	< 0.001	-0.45	-4.87	< 0.001	-0.77	-7.38	< 0.001
ps2 (middle-aged)	-0.35	-4.07	< 0.001	-0.25	-2.80	0.005	-0.45	-4.40	< 0.001
ps3 (elderly)	-1.01	-3.01	0.003	-1.12	-3.19	0.002	-0.91	-2.26	0.014
N / F / prob > F / R^2^	428 / 15.6 / < 0.001/ 0.10			428 / 9.0 / < 0.001/ 0.06			428 / 18.3 / < 0.001 /0.12		
	**V: Struggle for acceptance**			**Resistance**			**Acceptance**		
*Base outcome: Dying people*									
ps1 (young adults)	-0.72	-9.60	< 0.001	-0.38	-4.06	< 0.001	-1.05	-11.1	< 0.001
ps2 (middle-aged)	-0.88	-11.42	< 0.001	-0.71	-7.25	< 0.001	-1.08	-11.0	< 0.001
ps3 (elderly)	-0.44	-1.94	0.053	-0.14	-4.28	n.s	-0.80	-2.85	0.005
N / F / prob > F / R^2^	428 / 49.2 / < 0.001 / 0.29			428 / 20.6 / < 0.001 /0.14			428 / 53.0 / < 0.001/ 0.27		

**Coeff:** regression coefficient applying robust standard errors; **t** = t-value on t-distribution; **p** = level of significance of association; **ps1,2,3** = propensity score for compared subgroups by dying people as base outcome; **ps includes:** gender, education, migration background, parenthood, current partnership, any psychiatric hospitalization in lifetime, any psychiatric/psychotherapeutic treatment in lifetime, current intake of psychopharmacological, neuroticism, extraversion, openness, agreeableness, and conscientiousness

## Discussion

The higher scoring for all dimensions and factors in dying people compared with those for whom the end of life is an abstract future event, even old people not suffering from a (known) mortal disease, independent of the number of comorbidities, indicates that people who are facing the imminent end of their lives are more aware and confront themselves more intensively with dimensions derived from the theory of *Daseinsverabschiedung* (“Farewell to existence”, as anthropologically grounded). The coexistence of acceptance and resistance in assuming the end of life highlights the internal rifts and inner struggle in the face of death, not as a contradiction but as an ambivalence that has to be taken into account in the care of the dying.

The discussion section has to start with the justification and delimitation against other models. The "Anticipatory Farewell to Existence" construct is an anthropological one because it assumes the existence of pre-reflexive fundamental and constitutive dimensions of humans. As beings endowed with consciousness, humans are able to symbolise, first of all the external world and their own subjectivity as a self. One of the most relevant and dramatic symbolisations is the awareness of their own mortality that has be taken into account to orient their own finite existence. This investigation assumes the six philosophically justified fundamental dimensions that become genuine when an individual is confronted with his/her unavoidable finiteness due to a mortal disease. These fundamental dimensions are set a priori and cannot be empirically investigated, but justified philosophically, as in a previously mentioned philosophical work in German [12]. An empirical transfer means translating the dimensions and factors into a language of lifeworld experiencing and recording this experience in comprehensible questions that mean real experiences and reflections. This empirical translation is the AFEQT scale, which is based on cognitive achievements but refer to a pre-reflexive structure of human beings that can be reinforced by empirical investigations, but not definitively validated, because of its metaphysical nature. This is the main difference to other primarily psychologically-based models like death anxiety, mortality salience, coping with the threat of death, phases of dying, meaning of life and life attitudes when facing death, and finally, the concept of "anticipatory grief".

Elisabeth Kübler-Ross described in her monograph *Interviews with Dying Persons* [28] a theoretically elaborated model of the dying process as a sequence of different phases. This five-phase model is ultimately a typology that is not realised in its pure form in every dying person. The basic idea is that dying people run through a very special process that comprises phases. The difference between a staged model and the “Anticipatory Farewell to Existence” construct lies in the fact that there are no phases in the latter. End of life is understood as a period that is approached dialectically and not teleologically along the analytical dimensions of human existence and not psychologically in the sense of cognitions, emotions, motivations, values and personality. From a philosophical perspective, a justified critique of the psychological notion of dying as phasic has been made, especially because such models give the impression of normativity, namely that the dying process requires performance and, if not done, identifies a disorder that should restore "good dying". Gehring formulated this idea as follows: *"Is it (the setting of a time limit by phasing) merely a confrontation with a certain prognostic power that expects the responsible patient to see dying as a process and to take it upon himself in the sense of a ‘job’ to be done? The impression remains that psychology of the dying normalises and naturalises in certain way the personal confrontation with death. A new mixture of psychological needs and reason leads us to accept an objective modelling of ‘good death’"* ([29], pp. 184–185).

The concept of "anticipatory grief" proposed by Lindemann [30] predominantly refers to the relatives of dying persons both in the dying process and after death in order to better cope with illness by inwardly detaching themselves from their losses [31, 32]. At the end, it is about the production of meanings as giving sense to one’s own life. Assessment instruments have been developed, such as the "Anticipatory Grief Scale", which was recently psychometrically evaluated [33]. The concept of "Anticipatory Grief" differs from our construct of "Anticipatory Farewell to Existence" since the latter stresses the perspective of the person concerned, is not oriented towards coping strategies, does not assume a given overcoming, and renounces normativity.

Using a qualitative approach, Raoul and Rougeron identified dimensions of sense-making in people dealing with approaching the end of their life that are similar to those of AFE: reinterpretation of life, search for meaning, densification of the connection to the world, to loved ones, and to one’s self-control, vital energy, ambivalence towards the future, confrontation with death, and relationship with transcendence [34]. With the AFEQT questionnaire, we provide the first standardised instrument on an anthropological and not primarily psychological basis for the systematic investigation of this sense-making process with known psychometric properties [18]. Since the questionnaire is a formative and not reflective one, exploratory and confirmatory factor analyses are not appropriate.

The dimensions and factors of the AFE have to be considered as a theoretical framework that could provide orientation in sensitive dialogues with dying people in medical, nursing, and hospice settings, whereby the questions asked in the questionnaire are hints for developing supporting dialogues rather than compulsory questions to grasp the individual’s state of mind when facing death. The AFE may provide theoretical support to therapeutic interventions like “dignity therapy”, “spiritual care”, or “meaning-in-life” approaches, since meaning-in-life may positively influence not only personal attitudes but also medical symptoms in oncologic patients [35]. The result showing that 77% of dying participants reported a positive or very positive influence of interview questions on their reflections about existential issues at the end of life can be interpreted as meaning that the proposed anthropological dimensions are applicable for understanding confrontation with existential issues at the end of life, in particular the importance of interpersonality with relevant others.

As highlighted above, the dimensions proposed may be taken into account in supportive dialogues with dying people and their relatives, similarly to Dignity therapy [36], Meaning-centred psychotherapy [37], and Managing Cancer and Living Meaningfully (CALM) [38, 39], but also in the supervision of professionals working in palliative settings and in the self-reflection about the possible needs of patients along suggested anthropological dimensions, as in the Spiritual care approach [40]. Spiritual issues at the end of life include spiritual well-being, transcendence, hope, meaning and dignity [41], as well as forgiveness, self-exploration, search for balance, connection, self-actualisation, and consonance [42]. There are several points of overlap with meaning-in-life interventions that are associated with clinical benefits in measures of purpose-in-life, quality of life, spiritual well-being, self-efficacy, optimism, distress, hopelessness, anxiety, depression, and wish to hasten death [43]. Most of these issues can also be derived from the dimensions of AFE, indicating a convergence of all approaches that consider existential issues when facing end of life, because of their rooting in basic human needs.

The anthropological orientation of the *Daseinsverabschiedung* (AFE) theory outlined can be considered as its theoretical edge, implying a deeper comprehension of the human structure in the face of one’s own death that can also be translated into spiritual, moral, and psychological needs. Since the outlined dimensions and subdimensions are not intuitively set but derived from an anthropologically justified network of human constants, a broad and comprehensible approach to inner debates facing death is enabled, abstaining from normative expectations regarding existential tasks at the end of life. A further advantage of the AFE theory is its panoptic principle: on the one hand the valuing reminiscence of lived life; on the other hand the anticipation of possible circumstances around the own cessation-to-be towards grappling with remaining time [32, 44]. This panoptic principle considers and promotes the functions of reminiscence as portrayed in the subscales of the Reminiscence Functions Scale (RFS), especially to find biographical meaning and continuity, to maintain intimacy, to revive the bitterness of unlived life, and to prepare for death as an accepting stance [45], independent of assumptions about the sequential psychological phases of dying.

A gentle, unprejudiced, and respectful support of dying people can be seen as a humanistic mission when the concerns are rooted in the deepest human structure and the needs lead to a comprehensive attentiveness in care at the end of life [46, 47]. In a complementary inductive approach, a qualitative investigation of the personal constructions of meaning in relation to death would be of interest. In this way, relevant topics about confrontation with death would be generated from the free or low-structured narrative answers; however, this generalisation would have to be carried out separately for each homogeneous sample and, in a further step, the content analysis would have to be hermeneutically compared and illustrated with personal statements.

## Conclusion

Anthropological reflections on the transcendental structure of human beings, which is activated or actualised in a special way in the face of death, may provide a framework for practice towards the humanisation of medicine at the end of life, considering real experiences, possible needs, and underlying human conditions when confronted with one’s own death. The transfer of the “Anticipated Farewell to Existence” theory in medical practice at the end of life means first and foremost a stance that takes into account the basic structure of human confrontation with one’s own death in order to care sensitively and to assert individually, without moral prejudices or pressure regarding spiritual performances at the end of life and considering deep existential disharmony [48].

## Supplementary Information

**Additional file 1: Table 1.** Anticipatory Farewell to Existence Questionnaire (AFEQT). **Table 2.** Six-dimensional structure of the “Anticipatory Farewell to Existence” construct. **Table 3.** Descriptive statistics for each question of the “Anticipatory Farewell to Existence” Questionnaire. Assessment of group differences for each question.

## Data Availability

The authors provided all raw data on which the study is based. The Excel table with all raw data is provided in a supplementary file.

## Abbreviations

(1-β): Post hoc power. The power of a test is the probability of rejecting the null hypothesis –albeit a significant result – when the real difference is equal to the minimum effect size
I: Self-transcendence
Ia: Permanence
Ib: Metaphysical rise
II: Expiration of one’s existence time
IIa: Conclusion
IIb: Farewell
III: Altruistic preoccupation
IIIa: Bequest
IIIb: Charity
IV: Reconciliation with own existence
IVa: Fulfilment
IVb: Harmony
V: Struggle for acceptance
Va: Resistance
Vb: Acceptance
Α: Scale reliability coefficient
AFE: Anticipated Farewell to Existence
AFEQT: Anticipated Farewell to Existence Questionnaire
AIC: Average inter-item correlation
BFI-10: Big Five Inventory, 10 items
CALM: Managing Cancer and Living Meaningfully (Short psychotherapy)
CO: Coherence
Coef.: Robust regression coefficient
CR: Choice/Responsibleness
DA: Death acceptance
ECOG: Eastern Cooperative Oncology Group
ET: Existential transcendence
EU: Existential vacuum
F: Statistic of model
GS: Goal seeking
LAP-R: Life Attitude Profile-Revised
M: Mean
Md: Median (50th percentile)
N: Size of sample
n.s.: Not significant
PMI: Personal Meaning Index
PO-Bado: Psycho-Oncology ‒ Basic Documentation
Ps: Propensity score
PU: Purpose
R: Pearson correlation coefficient
R^2^: Explained variance of dependent variable
SD: Standard deviation
RFS: Reminiscence Functions Scale
Vc: Variation coefficient (SD/M) × 100
W: Value of Shapiro–Wilk statistics
