# Supplementary material for: Divergent patterns of confrontation with death using the Anticipated Farewell to Existence Questionnaire (AFEQT): a cross-sectional comparative study of four samples with increasing proximity to death
Source: BMC Palliat Care. 2021 Aug 8;20:125. doi: 10.1186/s12904-021-00818-y (PMC8349498; doi:10.1186/s12904-021-00818-y)
Supplement: Supplementary file 1 — Additional file 1: Table 1. Anticipatory Farewell to Existence Questionnaire (AFEQT). Table 2. Six-dimensional structure of the “Anticipatory Farewell to Existence” construct. Table 3. Descriptive statistics for each question of the “Anticipatory Farewell to Existence” Questionnaire. Assessment of group differences for each question. [file 12904_2021_818_MOESM1_ESM.docx]

**Supplementary Tables and Figures**

**Supplementary Table 1: Anticipatory Farewell to Existence Questionnaire (AFEQT)**

| No. | Dim. | Question | Does not apply to me at all  (0) | Applies somewhat to me  (1) | Applies to me partially (2) | Quite true for me  (3) | Fully applicable to me  (4) |
| --- | --- | --- | --- | --- | --- | --- | --- |
| 1 | 4.a.1. | I believe that by and large my life is okay. |  |  |  |  |  |
| 2 | 4.b.1. | When I look back, I am satisfied with my life. |  |  |  |  |  |
| 3 | 2.a.1. | It is good that I am still alive. |  |  |  |  |  |
| 4 | 2.b.1. | I am about to say goodbye to the people I care about. |  |  |  |  |  |
| 5 | 3.a.1. | I think I've been important to some others around me so far |  |  |  |  |  |
| 6 | 3.b.1. | I am aware that my relatives and friends will one day be in this world without me. |  |  |  |  |  |
| 7 | 1.a.1. | It reassures me to know that something of me will remain in this world. |  |  |  |  |  |
| 8 | 1.b.1. | I believe that after my death I will continue to exist in some other form. |  |  |  |  |  |
| 9 | 5.a.1. | I can usually stand the thought of my finiteness. |  |  |  |  |  |
| 10 | 5.b.1. | I can recognise and accept that there have been difficulties in relationships in my life. |  |  |  |  |  |
| 11 | 4.a.2. | I stand by what I have done in life. |  |  |  |  |  |
| 12 | 4.b.2. | I am grateful for my life so far. |  |  |  |  |  |
| 13 | 2.a.2. | Every new day is a gift for me. |  |  |  |  |  |
| 14 | 2.b.2. | My former dreams and wishes are no longer so important to me. |  |  |  |  |  |
| 15 | 3.a.2. | I will leave meaningful things on this earth. |  |  |  |  |  |
| 16 | 3.b.2. | I find it easy to comfort my relatives and friends when they are sad because of me. |  |  |  |  |  |
| 17 | 1.a.2. | It reassures me to know that someone else will remember me. |  |  |  |  |  |
| 18 | 1.b.2. | I experience myself integrated into a higher dimension. |  |  |  |  |  |
| 19 | 5.a.2. | Sometimes I don’t want to know anything about anything, I try to ignore it as best I can. |  |  |  |  |  |
| 20 | 5.b.2. | I have come to terms with the fact that I have been hurt before. |  |  |  |  |  |
| 21 | 4.a.3. | I believe I have achieved something in my life. |  |  |  |  |  |
| 22 | 4.b.3. | I believe that I have lived my life as well as I could. |  |  |  |  |  |
| 23 | 2.a.3. | I can accept my foreseeable finiteness. |  |  |  |  |  |
| 24 | 2.b.3. | I am becoming more and more modest. |  |  |  |  |  |
| 25 | 3.a.3. | I was and still remain important for some people. |  |  |  |  |  |
| 26 | 3.b.3. | I can withdraw in favour of others. |  |  |  |  |  |
| 27 | 1.a.3. | I believe that something of mine continues to work in someone. |  |  |  |  |  |
| 28 | 1.b.3 | I can loosen the control over my existence. |  |  |  |  |  |
| 29 | 5.a.3. | I allow my thoughts to confront my real life. |  |  |  |  |  |
| 30 | 5.b.3. | In retrospect, I can see that difficulties have also got me further. |  |  |  |  |  |
| 31 | 4.a.4. | I believe that it was good to be born. |  |  |  |  |  |
| 32 | 4.b.4. | I can cope with the missed and failed things in retrospect. |  |  |  |  |  |
| 33 | 2.a.4. | It is okay that not all wishes in life come true. |  |  |  |  |  |
| 34 | 2.b.4. | My hitherto existence has been valuable. |  |  |  |  |  |
| 35 | 3.a.4. | There are people who are important to me. |  |  |  |  |  |
| 36 | 3.b.4. | Even today I still know how I can help if someone in my surroundings needs it. |  |  |  |  |  |
| 37 | 1.a.4. | I believe that occasionally someone will think of me even after my death. |  |  |  |  |  |
| 38 | 1.b.4. | I can accept that my body re-enters the cycle of nature. |  |  |  |  |  |
| 39 | 5.a.4. | I always try to come to terms with my fluctuating feelings. |  |  |  |  |  |
| 40 | 5.b.4. | I have come to terms with the fact that I can no longer handle some issues that I would have liked to have resolved. |  |  |  |  |  |
| 41 | 4.a.5. | If it were possible, I would choose the same life again. |  |  |  |  |  |
| 42 | 4.b.5. | I can accept that in my life not all my wishes have come true. |  |  |  |  |  |
| 43 | 2.a.5. | I can come to terms with the length of my life. |  |  |  |  |  |
| 44 | 2.b.5. | I believe I have satisfactorily arranged matters of importance to me. |  |  |  |  |  |
| 45 | 3.a.5. | It is fine that I was allowed to love other people. |  |  |  |  |  |
| 46 | 3.b.5. | It is easy for me to talk to those close to me about my farewell to life. |  |  |  |  |  |
| 47 | 1.a.5. | Sometimes in my life I have behaved in a way that has been good for other people. |  |  |  |  |  |
| 48 | 1.b.5. | I feel integrated into the cycle of whole life. |  |  |  |  |  |
| 49 | 5.a.5. | Distractions from my situation are welcome. |  |  |  |  |  |
| 50 | 5.b.5. | I trust that injuries I have caused to others will heal. |  |  |  |  |  |
| 51 | 5.a.6 | I tend to deny life memories. |  |  |  |  |  |

| **Supplementary Table 2: Six-dimensional structure of the “Anticipatory Farewell to Existence” construct** | |
| --- | --- |
| **Self-**  **centred** | Dimension I: **Reconciliation with own existence**  Factor 1: ***Fulfilment of existence***  Factor 2: ***Harmony*** |
|  | Dimension II: **The expiration of the time of existence**  Factor 1: ***Conclusion***  Factor 2: ***Farewell*** |
|  | Dimension III: **Struggle for acceptance**  Factor 1: ***Resistance***  Factor 2: ***Acceptance*** |
| **Self- decentred** | Dimension IV: **Wounded physical integrity**  Factor 1: ***Physical disability***  Factor 2: ***Corporality as presence*** |
|  | Dimension V: **Altruistic preoccupation**  Factor 1: ***Bequest***  Factor 2: ***Charity*** |
|  | Dimension VI: **Self-transcendence**  Factor 1: ***Permanence***  Factor 2: ***Metaphysical rise*** |
| *The six proposed dimensions of the construct “Anticipatory Farewell to Existence” and the corresponding, partially complementary factors are presented in tabular form. The six dimensions are divided into “self-centred” and “self-decentred” dimensions according to a superordinate aspect of “centring”.* | |

| **Supplementary Table 3:** **Descriptive statistics for each question of the “Anticipatory Farewell to Existence” Questionnaire. Assessment of group differences for each question.** | | | | | | | | | | | |
| --- | --- | --- | --- | --- | --- | --- | --- | --- | --- | --- | --- |
|  |  |  | **Young adults (1)** | | **Middle-aged**  **(2)** | | **Elderly people (3)** | | **Dying persons (4)** | | **Differences** |
| **D** | **F** | **Q** | **M (SD)** | **Md** | **M (SD)** | **Md** | **M (SD)** | **Md** | **M (SD)** | **Md** | **Scheffé test** |
| **Self-transcendence** | **Permanence** | 1a1 | 2.26 (1.41) | 3 | 2.26 (1.38) | 3 | 2.59 (1.59) | 3 | 3.32 (1.29) | 4 | 4 > 1, 2, 3 |
|  |  | 1a2 | 3.07 (1.29) | 4 | 2.29 (1.29) | 3 | 3.08 (1.29) | 4 | 3.64 (1.02) | 4 | 3 > 2; 4 > 1, 2, 3 |
|  |  | 1a3 | 2.78 (1.34) | 3 | 2.51 (1.22) | 3 | 1.13 (1.30) | 4 | 3.49 (1.13) | 4 | 3 > 1, 2; 4 > 1, 2 |
|  |  | 1a4 | 3.46 (0.91) | 4 | 3.47 (0.70) | 4 | 3.59 (0.92) | 4 | 3.91 (0.47) | 4 | 2, 3 > 1; 4 > 1, 2 |
|  |  | 1a5 | 3.41 (0.93) | 4 | 3.22 (0.88) | 3 | 3.50 (0.93) | 4 | 3.93 (0.39) | 4 | 3 > 1; 4 > 1, 2, 3 |
|  | **Metaphysical rise** | 1b1 | 2.10 (1.61) | 2 | 2.78 (1.46) | 3 | 2.41 (1.62) | 3 | 2.33 (1.79) | 3 | 2 > 1 |
|  |  | 1b2 | 1.77 (1.65) | 1 | 2.63 (1.54) | 3 | 2.87 (1.58) | 4 | 2.66 (1.73) | 4 | 2, 3, 4 > 1 |
|  |  | 1b3 | 2.60 (1.32) | 3 | 2.14 (0.96) | 2 | 2.65 (1.20) | 3 | 3.31 (1.22) | 4 | 3 > 1, 2; 4 > 1, 2, 3 |
|  |  | 1b4 | 3.32 (1.16) | 4 | 3.18 (1.12) | 4 | 3.28 (1.29) | 4 | 3.34 (1.36) | 4 | ø |
|  |  | 1b5 | 3.16 (1.11) | 4 | 3.05 (0.95) | 3 | 3.33 (1.14) | 4 | 3.70 (0.91) | 4 | 3 > 1; 4 > 1, 2 |
| **Expiration of existence** | **Conclusion** | 2a1 | 3.26 (1.25) | 4 | 3.82 (0.46) | 4 | 2.68 (1.45) | 3 | 2.89 (1.52) | 4 | 3, 4 < 1, 2 |
|  |  | 2a2 | 2.84 (1.32) | 3 | 3.27 (1.02) | 4 | 2.93 (1.38) | 4 | 3.13 (1.44) | 4 | 2, 4 > 1 |
|  |  | 2a3 | 3.17 (1.25) | 4 | 3.10 (1.03) | 3 | 3.33 (1.21) | 4 | 3.50 (1.21) | 4 | 4 > 1 |
|  |  | 2a4 | 3.32 (1.04) | 4 | 3.25 (0.90) | 3 | 3.52 (0.94) | 4 | 3.89 (0.57) | 4 | 2, 3 > 1; 4 > 1, 2 |
|  |  | 2a5 | 3.01 (1.29) | 4 | 2.86 (1.03) | 3 | 3.55 (0.98) | 4 | 3.24 (1.43) | 4 | 3 > 1, 2 |
|  | **Farewell** | 2b1 | 1.24 (1.62) | 0 | 0.41 (0.80) | 0 | 0.36 (0.87) | 0 | 2.29 (1.75) | 3 | 4 > 1, 2, 3 |
|  |  | 2b2 | 2.42 (1.42) | 2 | 1.90 (1.54) | 2 | 2.93 (1.33) | 3 | 3.27 (1.25) | 4 | 3, 4 > 1, 2 |
|  |  | 2b3 | 2.59 (1.37) | 3 | 2.24 (1.18) | 2 | 3,05 (1,19) | 3 | 3.38 (1.22) | 4 | 3, 4 > 1, 2 |
|  |  | 2b4 | 3.32 (1.05) | 4 | 3.37 (0.79) | 4 | 3.36 (0.95) | 4 | 3.69 (0.85) | 4 | 2 > 1; 4 > 1, 2 |
|  |  | 2b5 | 2.85 (1.26) | 3 | 2.55 (0.98) | 3 | 3.52 (0.89) | 4 | 3.57 (1.02) | 4 | 3, 4 > 1, 2 |
| **Altruistic preoccupation** | **Bequest** | 3a1 | 3.15 (1.04) | 4 | 2.96 (0.88) | 3 | 2.72 (1.23) | 3 | 3.56 (0.94) | 4 | 4 > 1, 2, 3 |
|  |  | 3a2 | 2.81 (1.28) | 3 | 2.56 (1.14) | 3 | 2.62 (1.48) | 3 | 3.45 (1.16) | 4 | 4 > 1, 2, 3 |
|  |  | 3a3 | 3.36 (0.94) | 4 | 3.17 (0.78) | 3 | 2.81 (1.29) | 3 | 3.68 (0.89) | 4 | 4 > 1, 2, 3 |
|  |  | 3a4 | 3.87 (0.52) | 4 | 3.93 (0.24) | 4 | 3.77 (0.71) | 4 | 3.96 (0.38) | 4 | ø |
|  |  | 3a5 | 3.73 (0.67) | 4 | 3.80 (0.50) | 4 | 3.74 (0.79) | 4 | 3.97 (0.21) | 4 | 2, 4 > 1 |
|  | **Charity** | 3b1 | 3.39 (1.05) | 4 | 3.48 (0.88) | 4 | 3.56 (1.04) | 4 | 3.97 (0.22) | 4 | 2, 3 > 1; 4 >1, 2 |
|  |  | 3b2 | 2.42 (1.45) | 3 | 2.12 (1.01) | 2 | 2.45 (1.47) | 3 | 2.74 (1.60) | 4 | 4 > 1, 2 |
|  |  | 3b3 | 3.35 (0.91) | 4 | 3.02 (0.80) | 3 | 3.40 (0.86) | 4 | 3.70 (0.76) | 4 | 3 > 2; 4 > 1, 2 |
|  |  | 3b4 | 3.25 (1.13) | 4 | 3.33 (0.79) | 3 | 3.25 (1.20) | 4 | 3.47 (1.19) | 4 | 4 > 1 |
|  |  | 3b5 | 2.26 (1.56) | 3 | 2.13 (1.32) | 2 | 2.59 (1.53) | 3 | 2.71 (1.61) | 4 | 3 > 1; 4 > 1, 2 |
| **Reconciliation w. existence** | **Fulfilment** | 4a1 | 3.15 (1.15) | 4 | 3.21 (0.81) | 3 | 2.73 (1.57) | 3 | 3.24 (1.33) | 4 | 4 > 3 |
|  |  | 4a2 | 3.55 (0.70) | 4 | 3.35 (0.64) | 3 | 3.54 (0.85) | 4 | 3.85 (0.53) | 4 | 4 > 1, 2, 3 |
|  |  | 4a3 | 3.08 (1.12) | 3 | 2.96 (0.89) | 3 | 3.29 (1.20) | 4 | 3.90 (0.42) | 4 | 2, 3 > 1; 4 > 1, 2, 3 |
|  |  | 4a4 | 3.37 (1.10) | 4 | 3.56 (0.91) | 4 | 3.39 (0.94) | 4 | 3.45 (1.09) | 4 | ø |
|  |  | 4a5 | 2.61 (1.31) | 3 | 2.65 (1.01) | 3 | 2.55 (1.24) | 3 | 2.63 (1.37) | 3 | ø |
|  | **Harmony** | 4b1 | 2.98 (1.14) | 3 | 3.08 (0.83) | 3 | 2.90 (1.19) | 3 | 3.22 (1.19) | 4 | 4 > 1 |
|  |  | 4b2 | 3.54 (0.88) | 4 | 3.64 (0.69) | 4 | 3.15 (1.25) | 4 | 3.66 (0.89) | 4 | 2, 4 > 3 |
|  |  | 4b3 | 2.96 (1.23) | 3 | 2.95 (0.84) | 3 | 3.29 (1.10) | 4 | 3.75 (0.78) | 4 | 2, 3 > 1; 4 > 1, 2, 3 |
|  |  | 4b4 | 3.00 (1.23) | 3 | 2.82 (0.95) | 3 | 3.17 (1.21) | 4 | 3.50 (1.05) | 4 | 3 > 1; 4 > 1, 2 |
|  |  | 4b5 | 3.23 (1.04) | 4 | 3.10 (0.96) | 3 | 3.62 (0.82) | 4 | 3.81 (0.67) | 4 | 2, 3, 4 > 1;3, 4 >2 |
| **Struggle for acceptance** | **Resistance** | 5a1 | 3.04 (1.20) | 4 | 3.13 (0.98) | 3 | 3.09 (1.42) | 4 | 3.38 (1.20) | 4 | 4 > 1 |
|  |  | 5a2 | 2.23 (1.50) | 2 | 1.32 (1.20) | 1 | 1.80 (1.64) | 2 | 2.37 (1.77) | 3 | 1 > 2; 4 > 2 |
|  |  | 5a3 | 3.34 (0.88) | 4 | 2.96 (0.94) | 3 | 3.35 (0.92) | 4 | 3.71 (0.73) | 4 | 3 > 2; 4 > 1, 2 |
|  |  | 5a4 | 3.05 (1.12) | 3 | 2.35 (1.27) | 3 | 2.98 (1.21) | 3 | 3.72 (0.83) | 4 | 3 > 2; 4 >1, 2, 3 |
|  |  | 5a5 | 2.63 (1.32) | 3 | 1.76 (1.20) | 2 | 2.76 (1.16) | 3 | 3.04 (1.39) | 4 | 1, 3 > 2; 4 > 1, 2 |
|  |  | 5a6 | 0.97 (1.23) | 0 | 0.92 (1.07) | 1 | 1.20 (1.52) | 0 | 0.52 (1.11) | 0 | 2 < 1; 4 < 1, 2, 3 |
|  | **Acceptance** | 5b1 | 3.39 (1.00) | 4 | 3.03 (0.91) | 3 | 3.11 (1.27) | 4 | 3.83 (0.66) | 4 | 4 > 1, 2, 3 |
|  |  | 5b2 | 2.99 (1.24) | 3 | 1.98 (1.29) | 2 | 2.79 (1.37) | 3 | 3.77 (0.65) | 4 | 1 > 2; 4 > 1, 2, 3 |
|  |  | 5b3 | 3.41 (0.99) | 4 | 3.32 (0.88) | 4 | 2.88 (1.47) | 4 | 3.53 (1.09) | 4 | 4 > 3 |
|  |  | 5b4 | 2.36 (1.51) | 2 | 1.66 (1.35) | 2 | 2.53 (1.52) | 3 | 3.28 (1.42) | 4 | 3 > 1, 2; 4 > 1, 2, 3 |
|  |  | 5b5 | 3.14 (1.12) | 4 | 2.79 (0.91) | 3 | 3.32 (1.17) | 4 | 3.83 (0.61) | 4 | 3 > 1, 2; 4 > 1, 2, 3 |
| **D**=dimension; **F**=factor; **Q**=question; **M**=mean; **SD**=standard deviation; **Md**=median (percentile 50) | | | | | | | | | | | |
